# The redox-sensitive R-loop of the carbon control protein SbtB contributes to the regulation of the cyanobacterial CCM

**DOI:** 10.1038/s41598-024-58354-7

**Published:** 2024-04-03

**Authors:** Oliver Mantovani, Michael Haffner, Peter Walke, Abdalla A. Elshereef, Berenike Wagner, Daniel Petras, Karl Forchhammer, Khaled A. Selim, Martin Hagemann

**Affiliations:** 1 Department of Plant Physiology, Institute of Biosciences, University of Rostock, A.-Einstein-Str. 3, 18059 Rostock, Germany; 2 Interfaculty Institute of Microbiology and Infection Medicine Tübingen, University of Tübingen, Tübingen, Germany; 3 Chemistry of Natural and Microbial Products Department, Pharmaceutical and Drug Industries Research Institute, National Research Centre, Giza, Egypt; 4 Department of Protein Evolution, Max Planck Institute for Biology, Tübingen, Germany; 5 Institute of Biology, Microbiology/Molecular Physiology of Prokaryotes, University of Freiburg, Schänzlestraße 1, 79104 Freiburg, Germany; 6 Interdisciplinary Faculty, Department Life, Light and Matter, University of Rostock, Rostock, Germany

**Keywords:** Cyanobacteria, Carbon metabolism, CCM, Light acclimation, Redox regulation, PII superfamily, Signaling, Microbiology, Physiology

## Abstract

SbtB is a PII-like protein that regulates the carbon-concentrating mechanism (CCM) in cyanobacteria. SbtB proteins can bind many adenyl nucleotides and possess a characteristic C-terminal redox sensitive loop (R-loop) that forms a disulfide bridge in response to the diurnal state of the cell. SbtBs also possess an ATPase/ADPase activity that is modulated by the redox-state of the R-loop. To investigate the R-loop in the cyanobacterium *Synechocystis* sp. PCC 6803, site-specific mutants, unable to form the hairpin and permanently in the reduced state, and a R-loop truncation mutant, were characterized under different inorganic carbon (C_i_) and light regimes. Growth under diurnal rhythm showed a role of the R-loop as sensor for acclimation to changing light conditions. The redox-state of the R-loop was found to impact the binding of the adenyl-nucleotides to SbtB, its membrane association and thereby the CCM regulation, while these phenotypes disappeared after truncation of the R-loop. Collectively, our data imply that the redox-sensitive R-loop provides an additional regulatory layer to SbtB, linking the CO_2_-related signaling activity of SbtB with the redox state of cells, mainly reporting the actual light conditions. This regulation not only coordinates CCM activity in the diurnal rhythm but also affects the primary carbon metabolism.

## Introduction

Cyanobacteria evolved the inorganic carbon-concentrating mechanism (CCM) to adapt towards the declining availability of CO_2_ and to reduce the occurrence of the competing oxygenation reaction of ribulose-1,5-bisphosphate carboxylase/oxygenase (RuBisCO), the key enzyme of the Calvin–Benson–Bassham (CBB) cycle that is responsible for the fixation of CO_2_ in all oxygenic phototrophs^1,2^.

The main components of the cyanobacterial CCM consist of up to five uptake systems for inorganic carbon (C_i_, the sum of bicarbonate and CO_2_) including three bicarbonate transporters, namely SbtA, BicA and BCT1 complex, and the CO_2_ hydration system made up of the NDH1_3_ and NDH1_4_ complexes. Furthermore, the prokaryotic organelle carboxysome contains all active RuBisCO molecules and carbonic anhydrase (CA). The bicarbonate transporters significantly increase the intracellular bicarbonate concentration, which after entering the carboxysomes is converted by the CA into CO_2_, increasing its concentration around RuBisCO and thereby reducing the possibility of the enzyme to incorporate O_2_. The excess CO_2_ that escapes the carboxysomes and the environmental CO_2_ that permeates inside the cells through the membrane is converted to HCO_3_^-^ by the CO_2_ hydration system, re-supplying the bicarbonate pool^3,4^.

The CCM, apart from greatly reducing the rate of photorespiration, allows cyanobacteria to acclimate to fluctuating conditions, whether C_i_, light or else^5,6^. For this reason, the CCM needs to be tightly regulated. The expression of key CCM components especially C_i_ uptake systems is regulated at transcriptional level, whereas the activity regulation is mostly done by the carbon regulator protein SbtB. As member of the PII superfamily, the SbtB protein forms a homotrimer capable of regulating many components of the CCM and carbon metabolism. SbtB binds to different adenyl-nucleotides, which include ATP, ADP, AMP, cAMP, or c-di-AMP, and thereby can perceive various intracellular signals to modulate its interaction partners accordingly^7^. This is mainly achieved by its T-loop, a typical protein part characteristic of the PII superfamily, which can adopt different conformations depending on which nucleotide is found in the binding pocket and thereby modulating the interaction with various target proteins^8–10^.

Contrary to canonical PII proteins, SbtB can only bind adenyl nucleotides including the two second messengers cAMP and c-di-AMP^11,12^. Furthermore, SbtB presents features that are not found in the rest of the superfamily. One of which is its recently discovered apyrase activity, which causes the hydrolysis of ATP and ADP to AMP, thereby modulating the activity of SbtB. Another unique characteristic is the presence of the C-terminal redox-regulated loop (R-loop) in many cyanobacterial SbtBs, which can form a disulphide bridge and presumably acts as a redox-sensing structure^13^. In photoautotrophs, the light-driven photosynthetic electron transport reduces ferredoxin, which is mainly used to produce the reducing agent NADPH_2_ but can also reduce proteins via thioredoxins (Trx). Hence, the large fluctuations in the redox state of the cell in the diurnal light rhythm can be sensed by many proteins through the presence of redox-sensitive disulphide bridges that are often found in enzymes associated with photosynthesis or light regulation. The light-dependent activation of those proteins is usually mediated by the ferredoxin/Trx system, causing the reduction of cysteine pairs within the target protein in the light and their oxidation in the night^14,15^. In cyanobacteria the main form is TrxA, one of the proteins with the highest binding affinity for SbtB thereby most likely responsible for the reduction of the R-loop^13^. While the role of the R-loop in modulation of the CCM was not investigated yet, it was found to affect the apyrase activity, which is significantly decreased when the loop is unable to form a disulphide bridge and even more when truncated^13^.

The identified interaction partners of SbtB also include the bicarbonate transporter SbtA and the glycogen branching enzyme GlgB^12,16^. The SbtB-GlgB interaction is modulated through c-di-AMP binding to SbtB, which allows it to regulate glycogen turnover during diurnal rhythm. However, the primary target of SbtB is SbtA. Both proteins can interact in multiple manners depending on the nucleotide bound to SbtB. The binding of AMP or ADP promote a strong interaction between SbtB and SbtA, while ATP, cAMP or c-di-AMP hinder or prevent the binding of SbtB to SbtA^11,13,16,17^. In such a manner, SbtB can regulate SbtA activity by modulating bicarbonate transport when not needed by the cell^18^ and to function as a plug to prevent the back-flow of bicarbonate outside of the cells during darkness, to avoid waste of energy^19^.

Here, we performed an in-depth investigation into the function of the R-loop in *Synechocystis* sp. PCC 6803 (hereafter *Synechocystis*), in regard to its role as redox-sensing module, its impact on the modulation of the CCM and carbon metabolism, the binding affinity of SbtB to the different adenyl nucleotides and the affinity of SbtB to SbtA.

## Results

### The R-loop affects the binding affinity of nucleotides to SbtB

It was previously shown that the C-terminal loop of SbtB acts as a redox sensor to modulate the apyrase activity of SbtB, where it strongly influences the T-loop conformation. The folded R-loop (oxidized) is therefore incompatible with the correct structuring of the T-loop. When the R-loop is reduced (unfolded) or absent, however, the T-loop becomes folded and wraps around ATP or ADP, protecting them from hydrolysis. By contrast the oxidized R-loop prevents the protecting conformation of the T-loop, inducing the nucleotides-strained conformation and thereby exposing them to hydrolytic attack to form AMP^13^. Therefore, we assumed that the redox-state of the R-loop could additionally affect the nucleotide-binding affinities of SbtB. To gain deeper insights into the influence of the reduced R-loop on the ligand binding properties of SbtB, we performed isothermal titration calorimetry (ITC) experiments using recombinant SbtB variants either lacking the R-loop (Δ104) or mimicking its reduced state using alanine or serine substitutions of both Cys105 and Cys110 (C105A-C110A or C105S-C110S) (Suppl. Fig. S1). Both of the SbtB C105A-C110A and C105S-C110S variants were able to bind ATP with high affinity comparable to the oxidized wild-type (WT)-SbtB protein, while cAMP bound with a slightly lower affinity (Table 1). Notably, the binding of ADP and AMP were strongly impaired (Suppl. Fig. S1), yielding only very weak isotherm signals, for which the K_d_ values could not be calculated. Surprisingly, the truncated R-loop SbtB variant (Δ104) regained the ability to bind ADP and AMP and increased the binding affinity towards ATP as opposed to cAMP (Table 1). Those results show similarities to the nucleotide-binding properties of *Cyanobium* sp. PCC 7001 SbtB, which naturally lacks the R-loop^20^. To further confirm those findings, we created a single Cys substitution to serine (C105S or C110S) or deletion of the last Cys110 (SbtB-Δ109) and determined their binding affinities with ITC. Again, all those single point mutation variants showed a reduced ADP and AMP binding and preference for ATP and cAMP (Table 1). These results provided strong evidence for the SbtB R-loop to be an element influencing the binding affinities of adenyl-charged nucleotides, such as ATP, ADP and AMP, but not cAMP, in addition to modulating the apyrase activity.

**Table 1:** Dissociation constants (K_d_) as mean values in (µM) ± SD (n = 3) for the adenyl nucleotides ATP, ADP, AMP, and cAMP to SbtB variants with exchanged cysteine residues (C105A-C110A, C105S-C110S, C105S, S110S) or truncated R-loop (Δ109, Δ104). Statistical significant differences compared to SbtB WT values are denoted with asterisks (* p < 0.05; very weak binding was assumed to be less than 10 µM). UND: undetermined.

	*SbtB* WT (oxidized) (calculated from^11^)		*SbtB*-C105A-C110A		*SbtB*-C105S-C110S		*SbtB*-C105S		*SbtB*-C110S		*SbtB*-Δ109		*SbtB*-Δ104	
	Binding	K_d_ (µM)	Binding	K_d_ (µM)	Binding	K_d_ (µM)	Binding	K_d_ (µM)	binding	K_d_ (µM)	binding	K_d_ (µM)	Binding	K_d_ (µM)
ATP	Binding	62.5 ± 16.6	Binding	29.8 ± 14.1*	Binding	47.6 ± 21.3	Binding	40.5 ± 75.9	Binding	60.1 ± 6.7	Binding	32.5 ± 17.9	Binding	35.0 ± 7.1*
ADP	Binding	55.8 ± 7.7	very weak Binding*	UND	very weak Binding*	UND	very weak Binding*	UND	very weak Binding*	UND	very weak Binding*	UND	Binding	79.2 ± 55.1
AMP	Binding	87.4 ± 3.7	very weak Binding*	UND	very weak Binding*	UND	very weak Binding*	UND	very weak Binding*	UND	very weak Binding*	UND	Binding	78.2 ± 6.0
cAMP	Binding	30.3 ± 5.0	Binding	106 ± 42.4*	Binding	52.8 ± 34.7	Binding	36.2 ± 46.8	Binding	53.2 ± 35.3	Binding	70.2 ± 28.9	Binding	68.1 ± 10.1*

**Figure 1:**
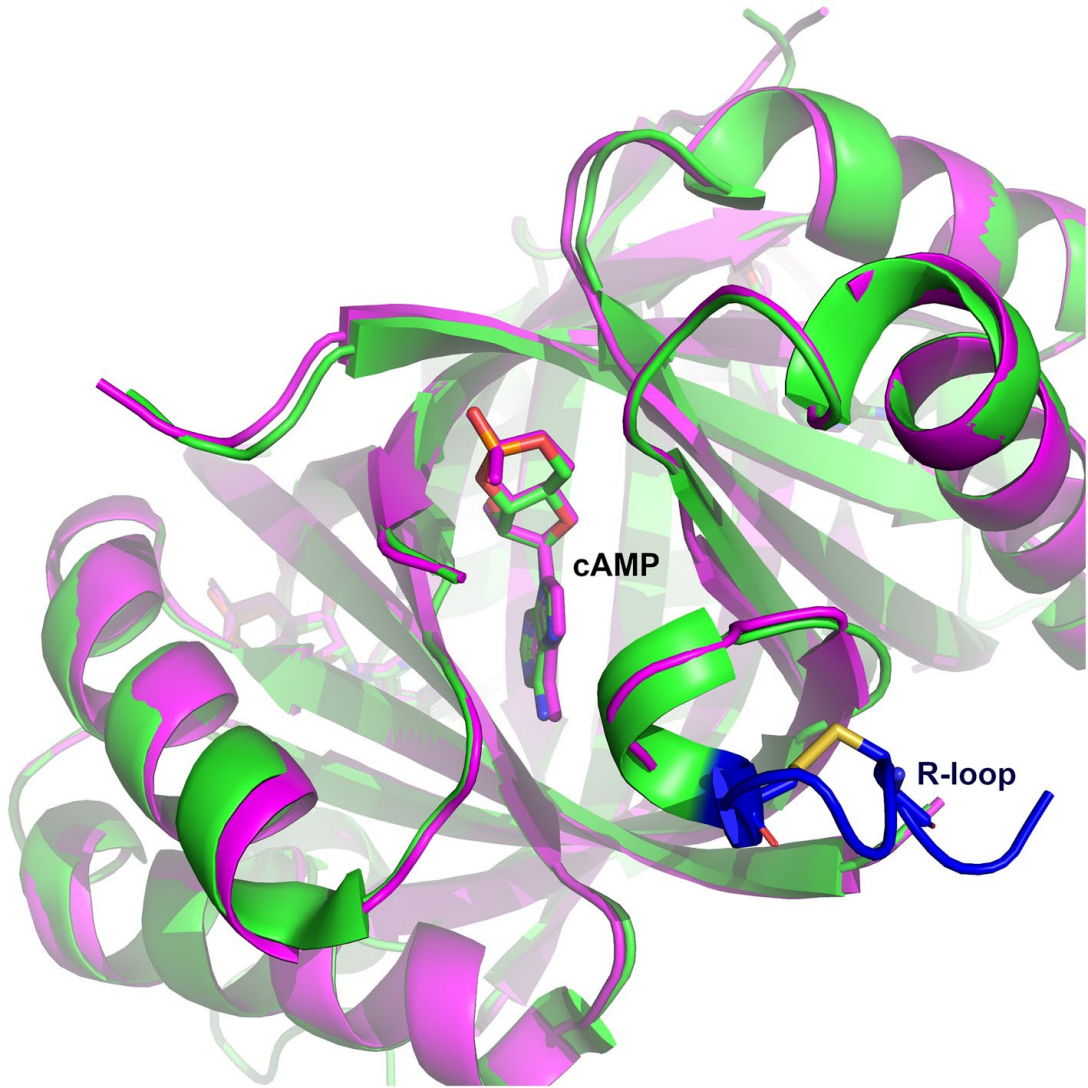
Reduced R-loop does not influence on cAMP binding. Alignment of oxidized *Synechocystis* WT-*SbtB*:cAMP (Green; PDB: 5O3Q) and *SbtB*-C105S-C110S:cAMP (Magenta) complex structures. cAMP molecules are visible in the middle of binding sites. The oxidized R-loop is highlighted in blue, with the disulfide bond in yellow.

To further support our results, we solved the crystal structure of the SbtB C105S-C110S variant in a complex with cAMP (Fig. 1), confirming its ability to retain a high affinity to cAMP, indicating that the reduced state of SbtB does not influence the binding of cAMP. The crystal space group of the C105S-C110S variant in a complex with cAMP was P4_1,_ similar to what was previously observed for the C105A-C110A variant in complex with ATP (PDB: 7R31), in which the R-loop was disordered^13^. In the structure of the SbtB C105S-C110S:cAMP complex, both T- and R-loops were disordered, resembling the *Anabaena variabilis* SbtB structure (PDB: 3DFE), showcasing a cAMP-binding mode almost identical to the previous structure of the oxidized *Synechocystis* WT-SbtB (PDB: 5O3Q)^11^. Since the structure did not provide further insights, it was not regarded further.

### The R-loop functions as a redox sensor

As all point mutation variants showed the same tendencies in respect to their changed sensory properties, we focused our efforts to obtain the *Synechocystis* R-loop mutants Δ*sbtB*::*sbtB*-C105A-C110A (hereafter C105A-C110A) and Δ*sbtB*::*sbtB*-Δ104 (hereafter Δ104), because these SbtB variants showed the least deviations in the nucleotide-binding assays (see Table 1). In mutant C105A-C110A the cysteines were replaced with alanine to simulate a permanent open structure of the reduced R-loop, whereas in Δ104 the entire R-loop was truncated. To further investigate the physiological role of redox sensing by SbtB through its R-loop, different growth experiments were performed with the R-loop mutants compared to Δ*sbtB* mutant.

**Figure 2:**
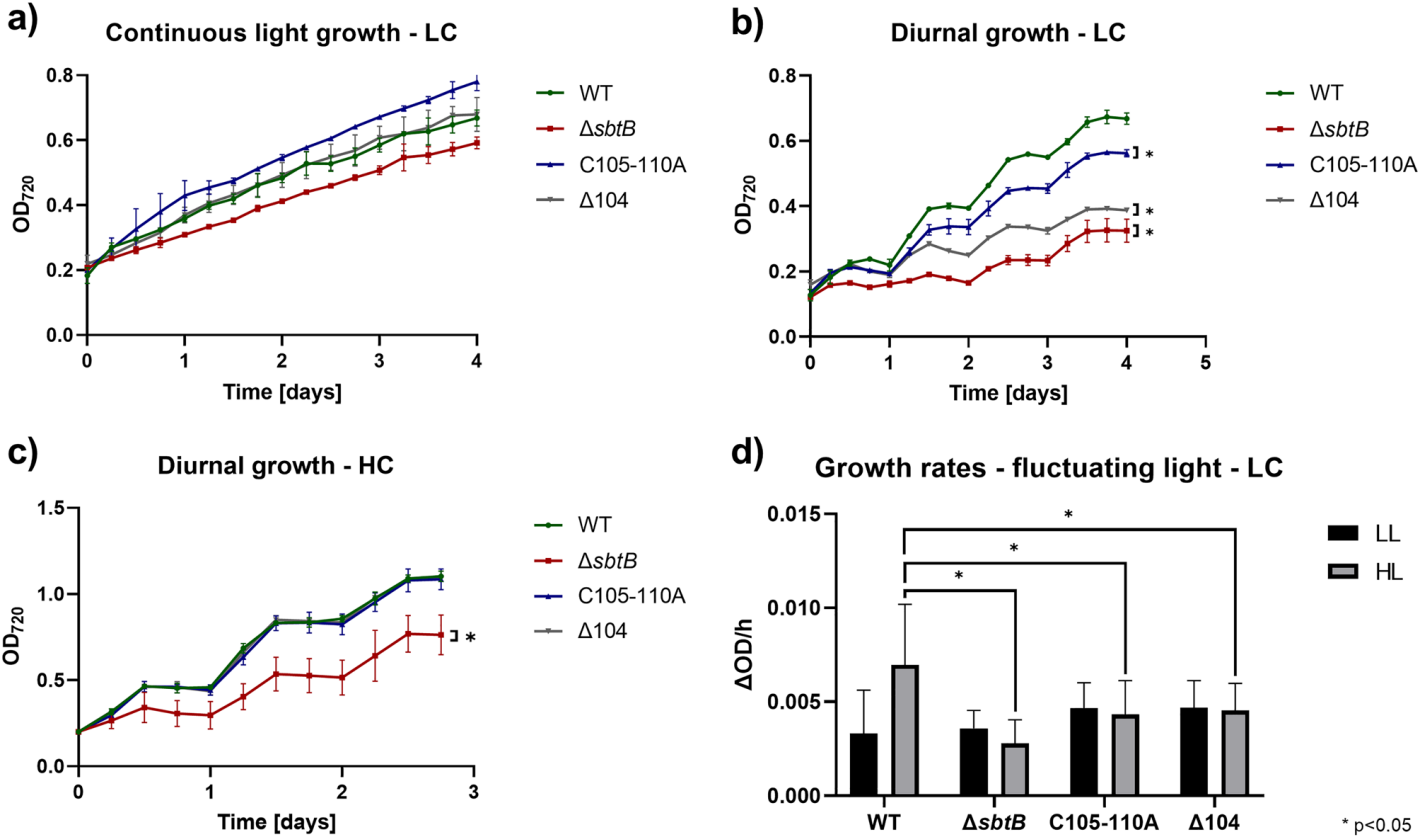
Impact of R-loop mutations on growth under different inorganic carbon (C_i_) and light conditions. Growth experiments using the *Synechocystis* WT strain and mutants Δ*sbtB*, C105A-C110A, and Δ104 were performed in the Multicultivator MD1000. (**a**) Growth under continuous light (130 µmol photons m^-2^ s^-1^) in low Ci (LC, ambient air of 0.04% CO_2_) conditions. (**b**) Growth in 12 h light (130 µmol photons m^-2^ s^-1^), 12 h darkness diurnal rhythm under LC conditions. (**c**) Growth in 12 h light (130 µmol photons m^-2^ s^-1^), 12 h darkness diurnal rhythm under high Ci (HC, air enriched with 5% CO_2_) conditions. (**d**) Growth rates (ΔOD_720_/h) under 6 cycles of fluctuating light conditions (LL: 3 h 100 µmol photons m^-2^ s^-1^, HL: 3 h 500 µmol photons m^-2^ s^-1^) at LC. Number of replicates (n) ≥ 3. * = Significance value (p) < 0.05.

The growth of these strains was first compared under ambient CO_2_ (0.04%, low C_i_, LC) and continuous light (Fig. 2a). Under these conditions, no significant growth difference was observed between the strains with mutated R-loop compared to WT, whereas the mutant Δ*sbtB* showed slower growth, consistent with our earlier finding^11^. The growth rate of the strains under high CO_2_ (5%, high C_i_, HC) conditions and continuous light was measured as well (Suppl. Fig. S2), showing again no significant difference. The relevance of the redox-sensing R-loop only became apparent, when the *sbtB* mutants were exposed to diurnal cycles (12 h day/12 h night) under LC (Fig. 2b) as well as HC (Fig. 2c) conditions. In the diurnal cycle, the photosynthesis and CCM activity needs to be activated when light turns on and is switched off upon onset of darkness. Under LC conditions, all *sbtB* mutants demonstrated a significantly impaired growth, with the most heavily impacted being Δ*sbtB*, followed by Δ104 and C105A-C110A, demonstrating that not only the presence of SbtB but also the redox sensing of the R-loop is relevant for acclimation to the diurnal rhythm. While under HC conditions, the R-loop mutants showed a growth similar to WT, the lack of SbtB still caused slower growth. Those phenotypes are likely caused by the influence of SbtB on glycogen metabolism^12,13^, indicating that the regulatory functions of SbtB during the diurnal cycle are not limited to those mediated by the redox-sensitive R-loop.

To further investigate the redox-sensing capability of the R-loop, the growth of the *sbtB* mutants was studied under fluctuating light with periods of standard growth at low light (3 h 100 µmol photons m^-2^ s^-1^) or high light (3 h 500 µmol photons m^-2^ s^-1^) in LC conditions (Fig. 2d). The WT strain experienced an approximately two-fold higher growth rate in high light (HL) periods than low light (LL) periods, whereas all *sbtB* mutants appeared to have lost the ability to acclimate to fluctuating light intensity, showcasing similar growth rates between HL and LL periods, with the Δ*sbtB* mutant performing the worst.

### The R-loop barely affects glycogen turnover during diurnal rhythm under HC conditions

**Figure 3:**
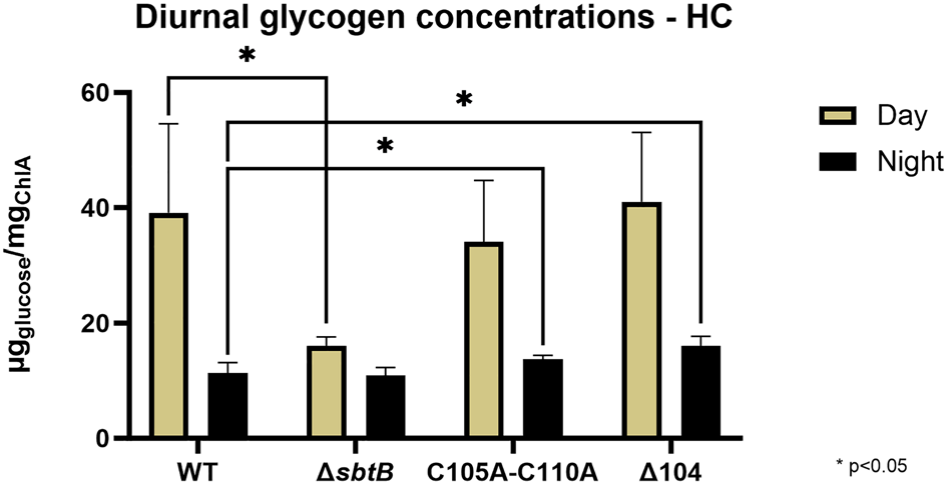
Effect of *sbtB* mutations on glycogen concentrations during diurnal rhythm at HC conditions. Glycogen concentration in the *Synechocystis* sp. PCC 6803 strains wild type (WT) and mutants Δ*sbtB*, C105A-C110A, or Δ104 were measured during growth under diurnal rhythm (12 h light, 12 h darkness) at high CO_2_ (5%, HC). Samples were taken in the middle of the day (Yellow) or night (Black) phases. n = 6. * p < 0.05.

As previously observed^11,13^, SbtB plays a direct role in controlling glycogen metabolism under diurnal conditions via interaction with the glycogen branching enzyme GlgB. The R-loop was also shown to be relevant in the interaction between SbtB and GlgB under LC conditions. To determine whether it is also important in the regulation of glycogen anabolism and/or catabolism under HC conditions, the glycogen concentrations in the cells of WT, Δ*sbtB* and R-loop mutant strains were determined during the light and dark phase of diurnal growth (Fig. 3).

The knock-out of *sbtB* was once again confirmed to greatly impact glycogen metabolism, causing a more than two-fold decrease in the glycogen concentration during the day and almost no glycogen breakdown during the night when compared to WT. The R-loop modulation of SbtB also appears to be slightly involved in the regulation of glycogen metabolism under HC conditions. The mutants accumulated similar amounts of glycogen during the day as WT cells, implying that under HC conditions, the R-loop does not influence glycogen synthesis. Consistent with the ability of SbtB R-loop variants to interact with GlgB^13^. However, less glycogen was consumed during the night, as the two R-loop mutants showed slightly higher glycogen concentrations during the dark phase compared to the WT cells. These findings indicate that the R-loop mutants are moderately affected in the glycogen catabolism.

### The R-loop influences the CCM activity

**Figure 4:**
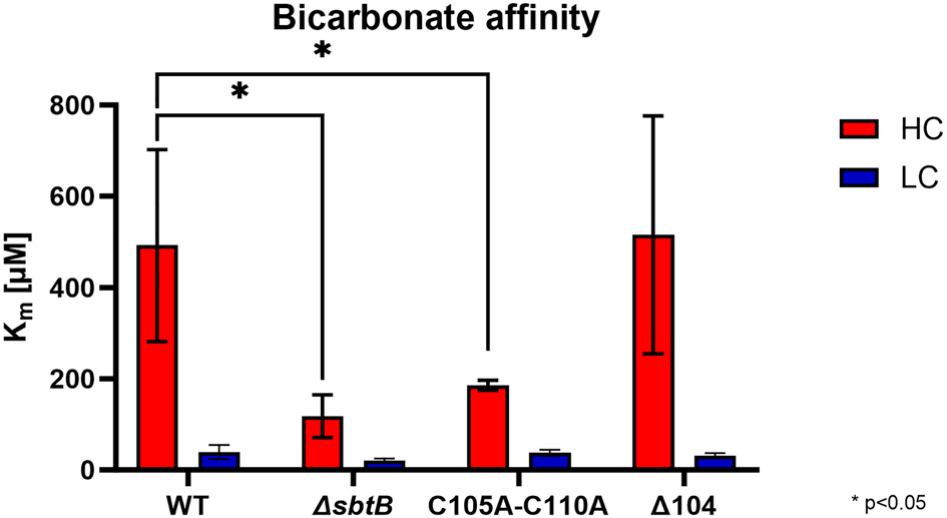
Impact of the *sbtB* R-loop mutations on CCM activity. Whole cell affinity to bicarbonate is expressed as average K_m_ values of µM bicarbonate of an oxygen evolution experiment using the *Synechocystis* strains wild type (WT), Δ*sbtB*, C105A-C110A, and Δ104. Cells were acclimated to either HC (Red) or LC (Blue) conditions. n ≥ 3. * p < 0.05.

In addition to glycogen turnover, SbtB has been shown to heavily impact many parts of the CCM^5^ and because of this, we have studied the effect of R-loop mutations on CCM activity by determining the bicarbonate-dependent oxygen evolution. To this end, the C_i_-dependent photosynthetic activity of WT, Δ*sbtB*, C105A-C110A, and Δ104 mutants was compared in cells acclimated to either HC or LC conditions. From the measured C_i_-dependent photosynthesis curves (Suppl. Fig. S3) the affinity to bicarbonate was calculated (Fig. 4).

As previously seen^5,11^, the knocking-out of *sbtB* caused a significantly higher affinity to C_i_ in cells grown under HC conditions, but comparable affinity under LC conditions. Surprisingly, the permanently open conformation mimicking the reduced state of the hairpin-loop in the C105A-C110A mutant caused a strong increase in the affinity to bicarbonate for HC-grown cultures, comparable to the Δ*sbtB* strain. Interestingly, the complete truncation of the R-loop (Δ104) reverted the bicarbonate affinity of HC cultures back to a WT-like state. Like the total absence of SbtB in the mutant Δ*sbtB*, changes in the R-loop had no significant impact on C_i_ affinity in cells acclimated to LC conditions.

### The TrxA reduces SbtB R-loop and thereby influencing the affinity of SbtB to SbtA

The discovery of the R-loop playing a role in the affinity of SbtB to second messengers caused us to re-evaluate the membrane association of SbtB under LC conditions as previously done for the native SbtB^11^. According to the results shown in Table 1, ADP and AMP are not bound to SbtB when the R-loop is reduced, for example during the day. This observation might indicate that the observed association of SbtB to SbtA under LC conditions could be affected by the oxidation of the R-loop.

**Figure 5:**
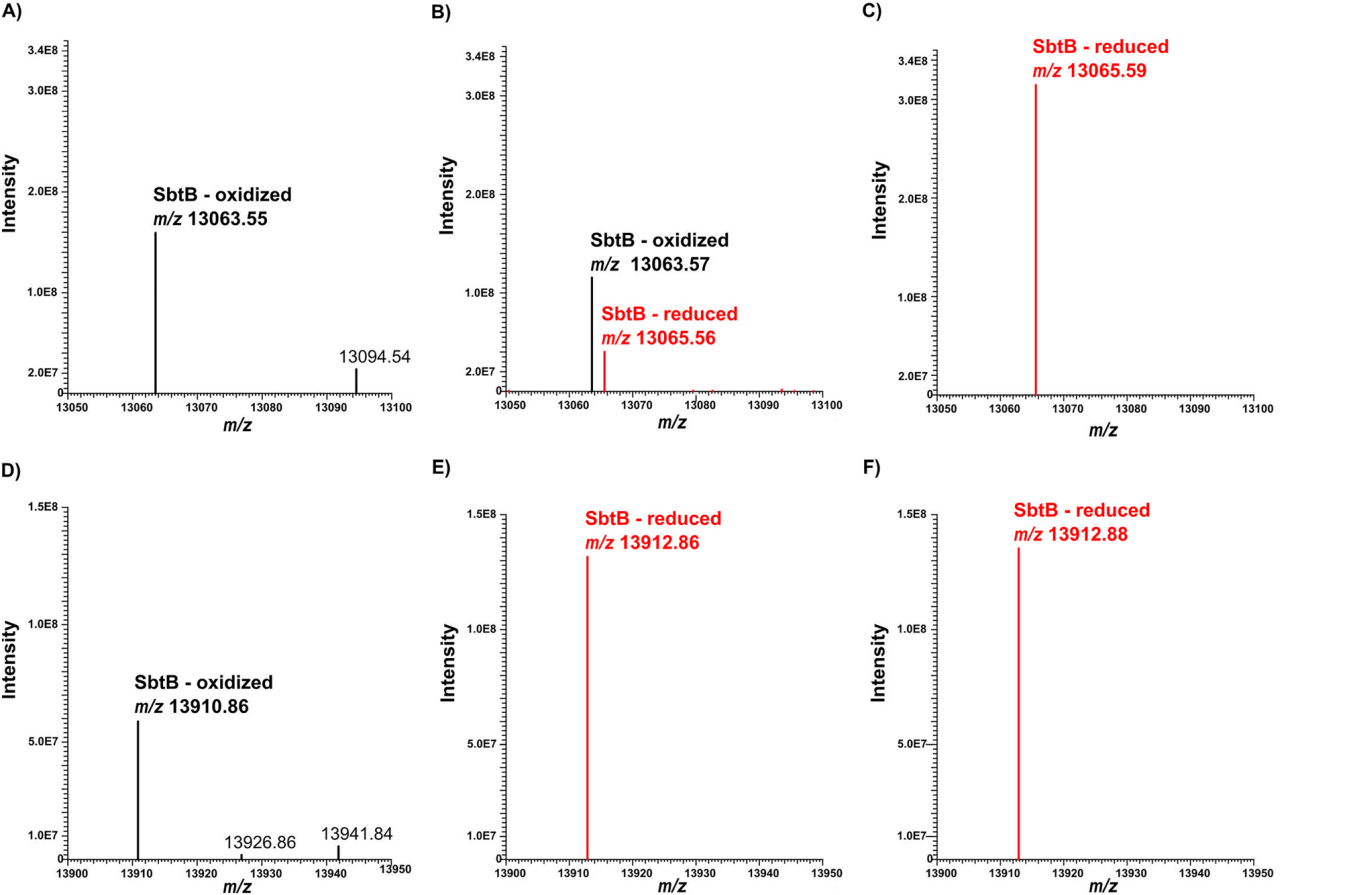
Intact protein mass spectrometry analysis indicating the redox status of *Synechocystis* SbtB without and after addition of reducing agents DTT and TrxA. Deconvoluted mass spectra of C-terminally (**A-C**) or N-terminally (**D-F**) tagged SbtB, either untreated (**A, D**), treated with 5 mM DTT (B, E), or with equimolar amounts of TrxA (**C, F**). Formation of a disulfide bond is labeled as “oxidized” and depicted in black. Reduction of the disulfide bond was observed as a mass shift by two protons (2 Da; depicted in red). C-terminal tagged SbtB treated with DTT was found in both redox states in the measurement displayed in panel **B** (also shown in Suppl. Figure S4).

We previously suggested that SbtB R-loop is reduced through an interaction with TrxA^13^, therefore we first determined the ability of TrxA to reduce SbtB through mass spectrometry (MS). Using a C-terminal Strep-tagged SbtB variant, intact protein MS analysis indicates that recombinant *Synechocystis* TrxA reduces SbtB, with a calculated mass of 13,065.59 Da, compared to the oxidized SbtB with mass of 13,063.55 Da (Fig. 5 and Suppl. Fig. S4). However, incubation of SbtB with the reducing agent DTT (5 mM) only led to a partial reduction of SbtB, suggesting that the C-terminal Strep-tag could provide extra protection for the R-loop. The experiment was therefore repeated using an N-terminal Strep-tagged SbtB variant, in which 5 mM DTT was able to efficiently reduce SbtB completely with a mass of 13,912.86 Da compared to the oxidized SbtB with 13,910.86 Da. To further confirm those results and determine the degree of SbtB reduction, we performed a Urea-PAGE experiment as described previously^21^. Incubation of equimolar concentrations of SbtB with DTT or TrxA in presence or absence of 5 mM DTT led to the appearance of a band shift towards the reduced state of SbtB. However, at lower DTT concentrations (0.5 mM), DTT alone was not able to reduce SbtB completely (Suppl. Fig. S5). These results further suggest the ability of TrxA to reduce SbtB more efficiently than other tested reducing agents. Collectively, these results imply firstly that TrxA is able to efficiently reduce SbtB even if the R-loop is protected, and secondly that in vivo*,* the native untagged SbtB would be more prone to reduction in response to the fluctuation of the redox state of the cell. Notably, similarly to SbtB, TrxA responds to the fluctuation of redox and diurnal state of the cell^22^.

**Figure 6:**
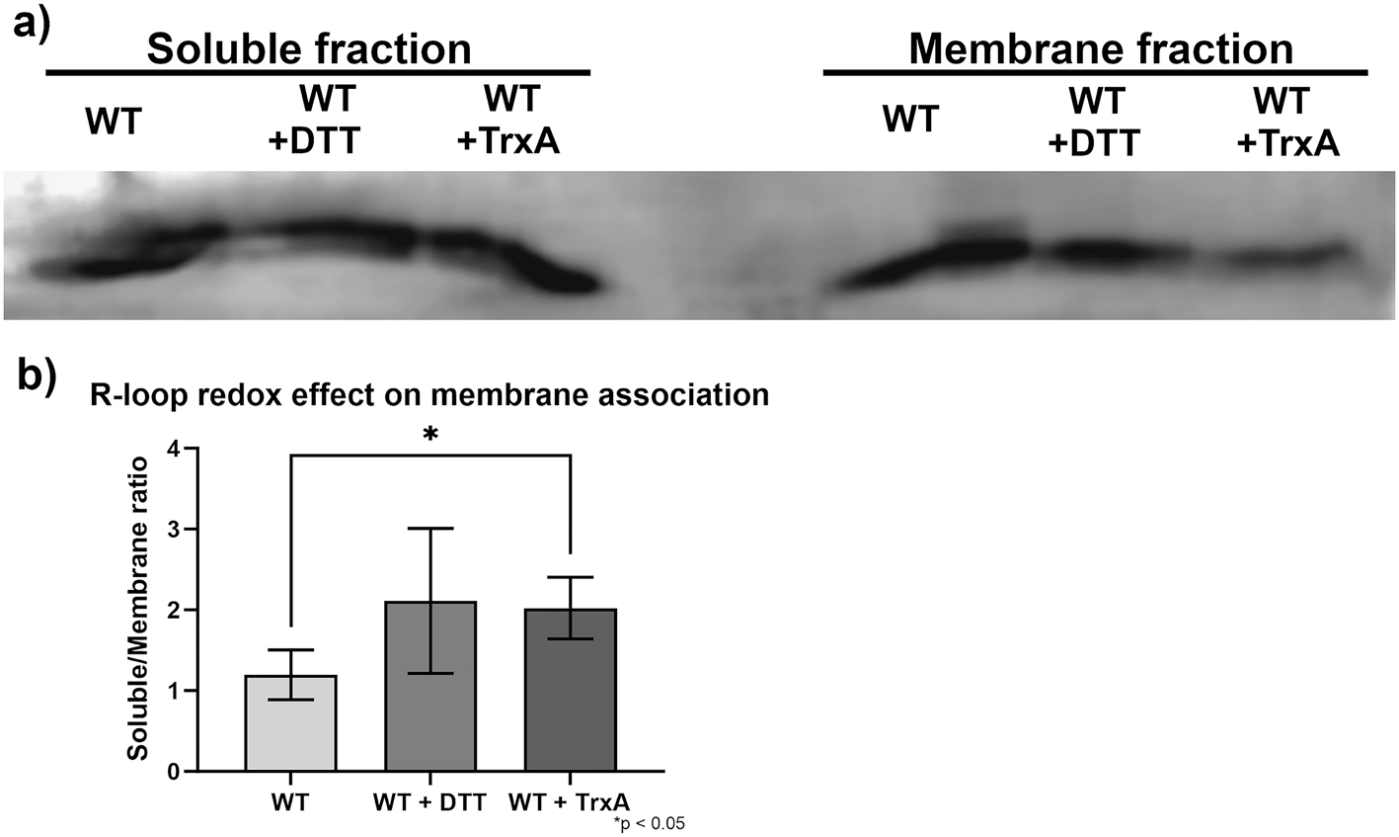
Impact of the redox state of SbtB on membrane association in Synechocystis. The membrane association of SbtB was determined by immune-blotting using an anti-SbtB antibody in two experiments in which soluble and membrane fractions were separated by high speed centrifugation. (**a**) Immuno-blot of membrane (M) and soluble (S) fractions of WT cells from LC conditions, either untreated, or incubated with either 5 mM DTT or equimolar TrxA. The full-size Western-blot is shown in Suppl. Fig. S6. (**b**) Ratio of relative membrane-associated SbtB to soluble in WT samples from immuno-blots shown in panel a). n = 3. * p < 0.05.

Thereafter, to investigate the membrane association of SbtB in *Synechocystis*, cell extracts of WT samples were subjected to immune-blotting using specific SbtB antibody after treatment with either 5 mM DTT or equimolar TrxA (Fig. 6a, the full-size Western-blot is shown in Suppl. Fig. S6). To this end, soluble and membrane fractions were compared to estimate the relative SbtB amounts in these fractions under oxidized or reduced conditions (Fig. 6b).

These experiments revealed that the treatment of the cell extracts with either DTT and especially TrxA caused a significant decrease in the amount of SbtB associated to the membrane, compared to the untreated samples. The difference was more pronounced in the TrxA-treated samples, showing once more its role in the reduction of SbtB. This result suggests that under oxidizing conditions, the membrane association of SbtB is clearly promoted, as the affinity of SbtB towards AMP would increase and induction of its apyrase activity, which altogether promote the membrane localization to SbtA.

### Truncation of R-loop affects the function of SbtB as a plug for SbtA

**Figure 7:**
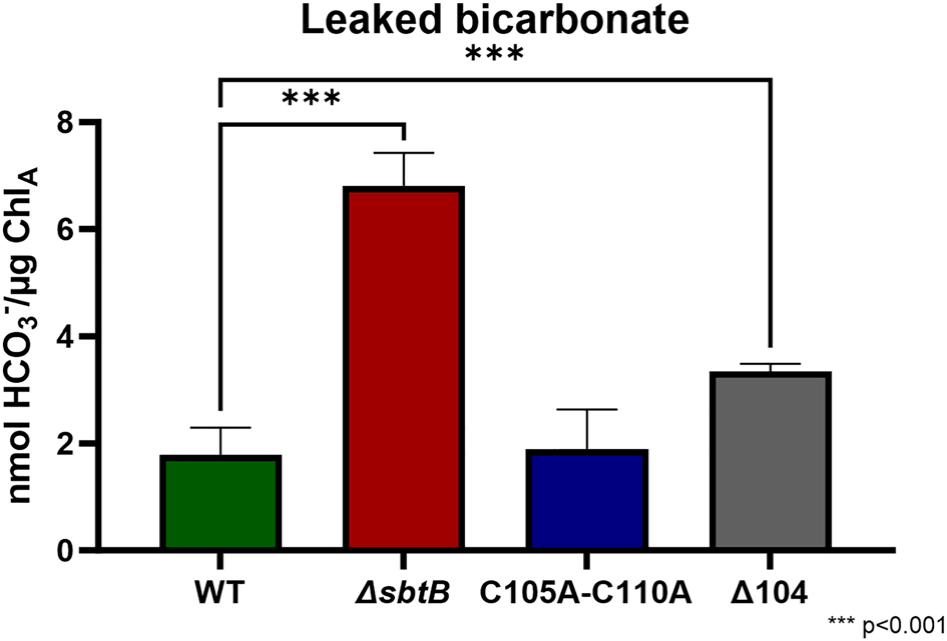
Role of the R-loop on the plug function of SbtB in *Synechocystis*. Concentration of radio-labelled bicarbonate per amount of Chlorophyll a leaked from cells of strains wild type (WT), Δ*sbtB*, C105A-C110A, and Δ104 is shown. n ≥ 6. *** p < 0.001.

Finally, as highlighted by Haffner et al*.*, 2023^19^, the interaction of SbtB with SbtA is not limited to the regulation of SbtA-mediated bicarbonate uptake, but presents more complex regulatory functions, such as plugging SbtA to prevent the leakage of bicarbonate when the CCM is not active. After discovering that mutations in the R-loop impact the affinity to adenyl nucleotides and/or the association of SbtB to the membrane, a C_i_ leakage experiment using ^14^C-labelled bicarbonate was performed (Fig. 7) to determine the influence of the R-loop of SbtB and the redox-state of the cell on the plug function of SbtB.

The experiment revealed, as seen before^19^, that the absence of SbtB causes a significantly higher amount of bicarbonate to be leaked during the dark incubation. The C105A-C110A mutation did not affect the leakage, whereas truncation of the R-loop in the Δ104 mutant caused a significantly increased leakage, albeit still almost two-fold inferior to what the knocking-out of *sbtB* caused.

## Discussion

During the last years, our and other groups provided evidence that the SbtB protein plays important roles in the regulation of the CCM and other aspects of the acclimation of the carbon metabolism to changing C_i_ amounts among cyanobacteria^7^. The structural investigations of SbtB^11–13,20^ and the super-complex of SbtB with SbtA^16,17^ showed that SbtB harbours different domains, which fulfil specific tasks such as binding of adenyl nucleotides and interaction with the bicarbonate transporter SbtA, whereas the frequently observed R-loop is probably involved in redox sensing. A bioinformatic investigation of 460 different cyanobacterial SbtBs revealed that the R-loop is present in roughly 50% of all SbtBs (Suppl. Fig. S7), with a high degree of conservation. It is always found at the C-terminal end of SbtB and contains the two conserved cysteines, which can form a disulphide bridge under oxidative conditions that significantly impacts the apyrase activity of SbtB^11,13^. Similar C-terminal extensions containing conserved cysteine pairs able to form disulphide bridges under oxidative conditions were added to specific enzymes of the CBB cycle (e.g., glyceraldehyde 3-phosphate dehydrogenase in chloroplasts) during evolution, which contributes to the Trx-mediated activation of the CBB in the light and its inactivation by oxidation during the night^15^. As in plants, the redox state in cyanobacteria is strictly correlated to the photosynthetic activity, it experiences large fluctuations between light or dark conditions, HC or LC conditions, and affects the activity of multiple components of the cell. This is achieved through proteins such as TrxA, the main Trx in cyanobacteria, which can regulate protein activities through the reduction of disulphide bridges, a common feature among different proteins involved in key metabolic processes such as gluconeogenesis and photosynthesis^14,23,27^. With its ability to bind TrxA^13^, the *Synechocystis* SbtB was a likely candidate for TrxA-mediated redox-regulation through its R-loop. This hypothesis that the redox state of the R-loop can coordinate for example SbtA-mediated Ci uptake and CBB cycle activity in diurnal light/dark cycles was investigated with a set of specific *Synechocystis* mutants in the present study.

To this end, we compared the Δ*sbtB* knock-out mutant with the site-specific mutants C105A-C110A and Δ104 of the model cyanobacterium *Synechocystis*. The proposed role of the R-loop as a redox-responsive module agrees with the impact of SbtB mutations on the general physiology of *Synechocystis* during acclimation to different light and C_i_ regimes, which are known to affect the cellular redox homeostasis in cyanobacteria as in eukaryotic phototrophs. Our results imply an important role of the R-loop in the regulation of the CCM during diurnal growth and moreover, under conditions of fluctuating light intensities. These phenotypic alterations were more pronounced, when cells were grown under LC conditions, when the CCM is more highly induced and active^5^. In contrast, under constant light conditions, which do not alter the cellular redox regime, the R-loop mutants behaved similar to WT (see Fig. 2, 3). The observed effect of the R-loop on glycogen synthesis under LC conditions^13^ could at least partially explain the observed growth phenotypes for the mutants, while measurement of glycogen concentrations during diurnal rhythm under HC conditions shows that the R-loop appears to play a more moderate role in the regulation of glycogen catabolism.

An oxygen evolution experiment provided insight into the role of the R-loop on SbtB’s regulation of the CCM as a whole under constant Ci conditions, revealing that the open conformation in the C105A-C110A mutants caused a Δ*sbtB*-like phenotype^5,11^, with significantly higher affinity to C_i_ for cultures acclimated to HC conditions. Surprisingly, the truncation of the R-loop in the Δ104 mutant caused the phenotype to revert to a WT-like state. This phenotype correlates with the loss of affinity of the SbtB variant C105A-C110A towards ADP and AMP, as the greatly reduced binding of these nucleotides would strongly impact the functionality of SbtB on the regulation of SbtA^16,17^ and the CCM^5^. This is further supported by the study of the truncated R-loop SbtB mutant Δ104, which displayed nucleotide affinities reverting to a WT-like state similar to those cyanobacteria that possess an SbtB without an R-loop, such as *Cyanobium* sp. PCC 7001^20^, with ATP showcasing the highest binding affinity, instead of cAMP as is the case for *Synechocystis*. Oxidation of the R-loop with subsequent generation of the SbtB-AMP state, which stimulates binding to SbtA, might promote the membrane associated state of SbtB. In agreement, we found diminished amounts of membrane-bound SbtB after treatment with reducing agents, especially TrxA, that very efficiently can convert the oxidized R-loop to the open reduced conformation in vitro. Thereafter, the role of the R-loop on the activity of SbtB as a plug for SbtA to prevent back-flow of bicarbonate outside the cells^19^ was investigated. Contrary to expectations, the open reduced R-loop conformation in mutant C105A-C110A did not seem to influence the leakage of bicarbonate, but its truncation caused more bicarbonate to flow out of the cells, possibly due to its smaller plug size. This result suggests that the binding of the different adenyl nucleotides is more relevant for the general regulatory properties of SbtB on the CCM, while in regard to its ability to plug SbtA, either the steric encumbrance is more relevant, or a still undiscovered regulation of SbtB comes into play.

Altogether, it became evident that R-loop is an extra element acquired during the evolution of SbtB to fine tune the nucleotide-binding affinity and to modulate the SbtB apyrase activity under diurnal conditions. The physiological meaning behind this regulation would be, during the day, for SbtB to always be in a reduced state and respond to fluctuation of C_i_ levels. Under LC, the increase in AMP levels could drive partial localization of SbtB to SbtA to prevent the back-flow of accumulated HCO_3_^-^^11,19^, while under HC conditions SbtB would remain localized in the cytoplasm. However, the system appears to also possess a “stand-by” state, where once it gets oxidized following sudden light changes, for example, the apyrase activity would promote the SbtA-B complex formation. But, the SbtB variants lacking the R-loop would only respond to changes in the adenylate charge, with drops in the ATP level for example during the night, shifting the equilibrium towards the SbtA-B complex. In agreement with this proposal, a recent study showed that the cyanobacterial SbtB proteins, lacking the R-loop, are responding mainly to energy state of the cell^18^. Therefore, the R loop addition made SbtB more versatile to integrate cellular energy and redox state than the basal SbtB without R-loop.

From our structural and ITC analysis of different R-loop variants, it also became obvious that the R-loop does not influence at all cAMP binding, but is only needed to fine tune the ADP/AMP affinity and regulate hydrolysis of ATP/ADP. Overall, this study provided evidence that the R-loop of SbtB provides an additional layer of regulation to the protein, which allows a finer regulation of the CCM in coordination with the entire photosynthetic C_i_ fixation for further adaptability to fluctuating conditions. However, the R-loop might not be the only component involved in the acclimation of SbtB structures and protein/protein interactions under different growth conditions as the protein possesses two amino acid residues that can accept a phosphate group through reversible protein kinase activity^9^. In fact, SbtB is a likely candidate of protein phosphorylation mediated by phytochromes, light receptors possessing kinase activity, which could add yet another layer of regulation to the protein, but this has yet to be investigated^24,25^.

## Materials and methods

### Strains and cultivation

The freshwater cyanobacterium *Synechocystis* sp. PCC 6803 was used as wild type (WT) for this study. The bacterium was cultured in BG11 media^26^ buffered with TES/KOH, at pH 7.0 for low-carbon (LC) and pH 8.0 for high-carbon (HC) conditions. LC cultures were bubbled with ambient air (0.04% CO_2_), while HC cultures with CO_2_-enriched air (5%, v/v). Growth on solid media was performed in plates with BG11 and added agar (10 g/L) at 28 °C and constant light of 30 µmol photons m^-2^ s^-1^. Growth in liquid media was performed in bubbling tubes at 29 °C with a light intensity of 130 µmol photons m^-2^ s^-1^. Dark incubation was performed with black bubbling tubes.

The *Synechocystis* mutant of the SbtB-encoding ORF *slr1513* (*sbtB* knock-out mutant, named Δ*sbtB*) harbors an erythromycin resistance, whereas the strains SbtB-C105A-C110A and SbtB-Δ104 possess resistance for spectinomycin. These strains were cultivated in the presence of 25 µg mL^-1^ erythromycin or 50 µg mL^-1^ spectinomycin.

### Generation of mutants

The *sbtB* mutants in *Synechocystis* have been obtained through homologous recombination. All constructs were generated using pUC19 plasmids, containing the modified *sbtB* coding regions (*slr1513*), a downstream spectinomycin resistance cassette, and the 500 base pairs up- and down-stream flanking regions necessary for recombination. The plasmids used for homologous recombination were obtained as described by^13^.

Cyanobacteria transformation was performed starting with inoculation of a WT culture at an OD ~ 0.5 then grown over night at LC conditions. The following day 10 mL of the culture were collected and centrifuged. The pellet was resuspended in 500 µL of fresh BG11 media, which was split in two new Eppendorf tubes, one for each transformation. 5 µg of the mutated-*sbtB*-containing pUC19 plasmid were added to each tube, which were then incubated in darkness on a shaker for 3 h at 28 °C. The transformed cultures were then plated on BG11 plates without antibiotics and grown for 3–5 days at 28 °C until the cell growth became visually apparent, after which a sterile water-spectinomycin (2.5 mg) 1:1 solution was added to each plate underneath the agar layer and the culturing was continued until the appearance of single colonies. The correct integration and segregation of the mutants was verified via PCR (2059 and 2092) and the correct coding sequence via sequencing using primer #PR6 (Suppl. Table S1).

### Growth rate determination

For the determination of growth rates, cultures were pre-acclimated to the desired carbon/light conditions for 3 days in bubbling tubes. Experiments started with inoculum at an OD_720_ of 0.2 in fresh BG11 media at pH 8.0 for HC or at pH 7.0 for LC Growth at different light and C_i_ regimes was then performed in Multicultivator MC-1000 (PSI, Czech Republic) that measures the OD_720_ every 15 min.

### Glycogen quantification

To determine concentrations of glycogen in *Synechocystis* strains during diurnal cycle, two independent cultures of each strain were started with an OD_750_ 1 under HC conditions and grown for 2 days under a 12 h light/12 h dark cycle in the Multicultivator MC-1000 with a light intensity of 100 µmol photons m^-2^ s^-1^. After the pre-acclimation, 5 mL samples were harvested in triplicates in the middle (6 h) of the light and dark phases, respectively, via centrifugation. Measurements of glycogen concentrations were performed as described previously^27^.

### Bicarbonate-dependent oxygen production

In vivo affinity for bicarbonate of HC- or LC-acclimated cultures of the *Synechocystis* strains used in this study was determined by the measurement of C_i_-dependent photosynthetic activity through a Clarke-type oxygen electrode (Hansatech). Cultures were re-suspended in fresh BG11 pH 8.0 medium at an OD_750_ = 3 (approximately 10 µg chlorophyll a per ml). Measurements were performed on 3 mL of the culture, illuminated with 300 µmol photons m^-2^ s^-1^ and increasing amounts of HCO_3_^-^. Oxygen evolution rates, normalized to the chlorophyll a content, were used to calculate the affinities to C_i_ of each strain as function of K_m_ values. K_m_ values were obtained from the linear regression of the bicarbonate/oxygen evolution curve, where V_max_ = 1/m and K_m_ = V_max_ * Y intercept.

### Heterologous expression and purification of TrxA protein

The pET15b plasmid carrying the gene for the His-Tagged TrxA, generated in Tübingen, was used for the heterologous expression and purification of TrxA. The *E. coli* strain carrying the plasmid was inoculated from an LB agar plate to 5 mL LB medium containing 50 µg/mL ampicillin, and grown over-night (ON) at 37 °C. The pre-culture was then used the following day for the inoculation of 200 mL LB medium without antibiotics and grown on a shaker at 37 °C until it reached an OD_600_ between 0.6 and 0.8. IPTG 50 µg/mL was then added for induction of protein expression and the culture was left in incubation on a shaker ON at room temperature (RT). Afterwards, the cells were collected by centrifugation for 15 min at 4 °C and 9000 rpm, then re-suspended in 5 mL homogenization buffer (20 mM Tris, 50 mM NaCl, pH 7.8). The cells were lysed with four cycles of sonication of 30 s with a BANDELIN sonoplus HD70 sonicator at 70% of the maximum output, interspersed with 30 s cooling on ice.

His-tagged purification was achieved via affinity chromatography with Ni–NTA column (Bio-Rad) as described previously^28^, pre-equilibrated with 5 mL homogenization buffer twice. After loading the lysate, collecting the flow-through, and washing the column twice with washing buffer (Tris 20 mM, NaCl 300 mM, pH 7.8), TrxA was eluted with elution buffer (Tris 20 mM, NaCl 500 mM, Imidazole 300 mM, DTT 2 mM, pH 7.8).

### Immuno-blotting for determining cellular localization of SbtB

Whole cell extracts were performed as in^11^, after adding 200 µL glass beads (Sigma) with a diameter of 465–600 µm, using a Retsch MM400 beads beater with a speed of 30 beats/s with 2 cycles of 30 s, interspersed with incubation on ice for 30 s. The membrane and soluble fractions were separated by centrifugation at 21,000 × g for 30 min at 4 °C. Total protein content was determined with the Roti-Nanoquant Bradford assay (Roth). 10 µg of total protein extracts were separated via SDS 15% poly-acrylamide gel electrophoresis, which were then transferred to PVDF membranes (BIO-RAD) via semi-dry blot transfer. The membranes were blocked with a 5% milk 1 × TBS buffer for 1 h and then incubated ON at 4 °C with the polyclonal anti-SbtB antibody (1:1000). Subsequently, the membranes were incubated with a goat anti-rabbit antibody coupled with a horseradish peroxidase (BIO-RAD) (1:5000). Images were developed by incubation with ECL Bright (Agrisera) and taken with a C-DiGit (LI-COR).

### Recombinant SbtB protein purification and Isothermal titration calorimetry (ITC)

All the plasmids and primers used in this study are listed in (Suppl. Table S1). The recombinant N- or C-terminal StrepII-tagged SbtB or it’s R-loop variants were expressed and purified on strep-tactin column (IBA) as described previously^11,13^. The ITC experiments were conducted as described previously^29,30^ using a MICROCal PEAQ-ITC SYSTEM (Malvern, UK) microcalorimeter or a VP-ITC microcalorimeter (MicroCal, LCC). Sample solutions were prepared by dialyzing the SbtB proteins, including SbtB-WT or its variants (SbtB-C105S, SbtB-C110S, SbtB-C105S-C110S, SbtB-C105A-C110A, SbtB-Δ104, and SbtB-Δ109), separately in a buffer of 50 mM Tris (pH 7.8), 150 mM KCl and 150 mM NaCl at 25 °C. Nucleotides (ATP, ADP, cAMP, and AMP) were used as ligand solutions. To ensure successful ITC runs, both protein and ligand solutions were prepared in the same buffer and condition to minimize heat changes arising from solution mismatches during mixing. Heat isotherms of the ligand dilution in the cell buffer were collected as blank runs for each experiment in the absence of protein.

For determining ligand binding isotherms of SbtB WT and its variants, 30 μM protein solutions (trimer concentration) were titrated against 1.5 or 2 mM of ligand solutions. The binding isotherms were calculated from the received data and fitted using one set of binding sites model with the MicroCal PEAG-ITControl Software (Malvern, UK) to calculate the dissociation constant K_D_. All titrations were performed in duplicates with different purification batches of recombinant proteins. The baseline subtraction and fitted offset were conducted based on the default control subtraction type of the software. The K_D_ value for the WT-SbtB protein was re-calculated from^11^ using a single binding model.

### Crystallization

The crystallization screens were done using vapor diffusion method in 96-well sitting-drop plates at 20 °C as described previously^11,13^. For co-crystallization of SbtB C105S-C110S variant with cAMP, 2 mM of cAMP was added to the protein solution. The co-crystal was obtained with a reservoir solution composed of 0.1 M MES pH 6.5 and 25% (w/v) PEG 1000. Our previous SbtB structure (PDB: 7R31) in space group P4_1_ was used as a search model and the structure was solved using difference Fourier method.

### Intact protein mass spectrometry analysis

For denaturing TopDown Proteomics analysis of SbtB redox state, 10 µM SbtB protein (calculated based on the monomeric molecular weight) either in presence or absence of 10 µM TrxA were incubated for 30 min at 28 °C in reaction buffer [50 mM Tris/HCl (pH 8.0); 250 mM NaCl; 0.5 mM EDTA] either without reducing agent or containing 5 mM DTT. Samples were shock frozen in liquid nitrogen and stored at -80 °C upon analysis. Intact protein MS analysis was carried out by injecting 5 µL of SbtB reaction mixture onto a Q Exactive HF hybrid quadrupole-Orbitrap mass spectrometer couple to a Vanquish UHPLC system (Thermo Scientific) using a Supleco C18 BioWide column (150 × 2 mm) with a flow of 0.4 mL/min and a linear gradient ramped over 5 min from 5 to 70% acetonitrile/water + 0.1% formic acid. MS1 and MS/MS spectra were acquired in Top3 data dependent acquisition mode. Resolution for both MS1 and MS/MS scans was set to 120,000 at 200 m/z. Data analysis was performed using Xcalibur 4.1 (Thermo Scientific). Deconvolution of the spectra was performed with Xtract using FreeStyle™ 1.8 SP2. Protein spectrum matching of the MS/MS was performed using *ProSight Lite*^31^.

### Urea-PAGE analysis

For denaturing urea PAGE analysis of the SbtB redox state, 10 µM reduced TrxA were incubated with 10 µM SbtB (calculated based on the monomeric molecular weight) in reaction buffer [50 mM Tris/HCl pH 8.0; 250 mM NaCl; 0.5 mM EDTA] at 28 °C for 30 min. As control, 10 µM SbtB were parallel incubated in reaction buffer that either contained 5 mM or 0.5 mM DTT or without reducing agent. All samples were mixed with and equal volume of two-fold concentrated non-reducing sample buffer [125 mM Tris–HCl, pH 6.8; 20% glycerol; 4% SDS; 0.1% bromophenol blue] and applied to a 14% 8 M urea-PAGE^21^. The urea-PAGE was pre-run in 1X TBE running buffer [10.8 g/l Tris; 5.5 g/l boric acid; 0.75 g/l EDTA] at 20 V for approx. 30 min, before samples were applied and run at 40 V. SbtB was subsequently identified by western blotting against SbtB, using a specific α-SbtB antiserum^11^.

### Bicarbonate leakage quantification

To determine the back-flow of ^14^C-labbelled HCO_3_^-^ from *Synechocystis*, each strain was acclimated to LC conditions for 2 days, then, on the day of the experiments, cultures were re-started at an OD_750_ = 1.0 in fresh BG11 pH 7.0 and grown for 2 h. Leakage was determines as described by Haffner et al*.*, 2023^19^.

### Statistical analysis

The statistical relevance of each result has been determined through an unpaired t Test via the two-stage step-up method^32^.

### Supplementary Information

Supplementary Information 1.

Supplementary Information 2.

Supplementary Information 3.

Supplementary Information 4.

## Data Availability

The raw data for the experiments of this work are available in the supplementary information. The raw and processed data from the intact protein MS experiments is available through the MassIVE repository (massive.ucsd.edu) with the identifier MassIVE MSV000090130.

## References

[1] Raven JA, Beardall J, Sánchez-Baracaldo P (2017). The possible evolution and future of CO_2_- concentrating mechanisms. J. Exp. Bot..

[2] Hagemann M (2016). Evolution of photorespiration from cyanobacteria to land plants considering protein phylogenies and acquisition of carbon concentrating mechanisms. J. Exp. Bot..

[3] Rae BD, Long BM, Badger MR, Price GD (2013). Functions. compositions. and evolution of the two types of carboxysomes: Polyhedral microcompartments that facilitate CO_2_ fixation in cyanobacteria and some proteobacteria. Microbiol. Mol. Biol. Rev..

[4] Hagemann, M., Song, S., & Brouwer, E. M. Inorganic carbon assimilation in cyanobacteria: Mechanisms, regulation, and engineering. In Hudson, P., Lee, S.Y., Nielsen, J. (Eds.), *Cyanobacteria Biotechnology, Wiley-Blackwell Biotechnology Series*, Chapter 1, 1–31 (2021).

[5] Mantovani O (2022). The impact of the cyanobacterial carbon-regulator protein SbtB and of the second messengers cAMP and c-di-AMP on CO_2_-dependent gene expression. New Phytol..

[6] Köbler C, Schultz S-J, Kopp D, Voigt K, Wilde A (2018). The role of the Synechocystis sp. PCC 6803 homolog of the circadian clock output regulator RpaA in day–night transitions. Mol. Microbiol..

[7] Mantovani O, Haffner M, Selim KA, Hagemann M, Forchhammer K (2023). Roles of second messengers in the regulation of cyanobacterial physiology: The carbon-concentrating mechanism and beyond. Microlife.

[8] Forchhammer K, Lüddecke J (2016). Sensory properties of the PII signalling protein family. FEBS J..

[9] Forchhammer K, Selim KA, Huergo LF (2022). New views on PII signaling: From nitrogen sensing to global metabolic control. Trends Microbiol..

[10] Forchhammer K, Selim KA (2020). Carbon/nitrogen homeostasis control in cyanobacteria. FEMS Microbiol. Rev..

[11] Selim KA, Haase F, Hartmann MD, Hagemann M, Forchhammer K (2018). PII-like signaling protein SbtB links cAMP sensing with cyanobacterial inorganic carbon response. Proc. Natl. Acad. Sci. USA.

[12] Selim KA (2021). Diurnal metabolic control in cyanobacteria requires perception of second messenger signaling molecule c-di-AMP by the carbon control protein SbtB. Science Advances.

[13] Selim KA (2023). Carbon signaling protein SbtB possesses atypical redox-regulated apyrase activity to facilitate regulation of bicarbonate transporter SbtA. Proc. Natl. Acad. Sci. USA.

[14] McFarlane CR (2019). Structural basis of light-induced redox regulation in the Calvin-Benson cycle in cyanobacteria. Proc. Natl. Acad. Sci. USA.

[15] Foyer CH, Noctor G (2009). Redox regulation in photosynthetic organisms: Signaling, acclimation, and practical implications. Antioxid. Redox. Signal..

[16] Liu XY (2021). Structures of cyanobacterial bicarbonate transporter SbtA and its complex with PII-like SbtB. Cell Discovery.

[17] Fang S (2021). Molecular mechanism underlying transport and allosteric inhibition of bicarbonate transporter SbtA. Proc. Natl. Acad. Sci. USA.

[18] Förster B, Mukherjee B, Rourke LM, Kaczmarski JA, Jackson CJ, Price GD (2023). Adenylnucleotide-mediated binding of the PII-like protein SbtB contributes to controlling activity of the cyanobacterial bicarbonate transporter SbtA. eLife.

[19] Haffner M (2023). PII signal transduction superfamily acts as a valve plug to control bicarbonate and ammonia homeostasis among different bacterial phyla. BioRxiv.

[20] Kaczmarski JA (2019). Structural basis for the allosteric regulation of the SbtA bicarbonate transporter by the PII-like protein, SbtB, from Cyanobium sp. PCC7001. Biochemistry.

[21] Ibrahim IM, Rowden SJL, Cramer WA, Howe CJ, Puthiyaveetil S (2022). Thiol redox switches regulate the oligomeric state of cyanobacterial Rre1, RpaA and RpaB response regulators. FEBS Lett..

[22] Pérez-Pérez ME, Martín-Figueroa E, Florencio FJ (2009). Photosynthetic regulation of the cyanobacterium *Synechocystis* sp. PCC 6803 thioredoxin system and functional analysis of TrxB (Trx x) and TrxQ (Trx y) thioredoxins. Mol. Plant.

[23] Schürmann P, Buchanan BB (2008). The ferredoxin/thioredoxin system of oxygenic photosynthesis. Antioxid. Redox Signal..

[24] Shin AY (2016). Evidence that phytochrome functions as a protein kinase in plant light signalling. Nat. Commun..

[25] Oren N, Timm S, Frank M, Mantovani O, Murik O, Hagemann M (2021). Red/far-red light signals regulate the activity of the carbon-concentrating mechanism in cyanobacteria. Sci. Adv..

[26] Rippka R, Deruelles J, Waterbury JB, Herdman M, Stanier RY (1979). Generic assignments. Strain histories and properties of pure cultures of cyanobacteria. J. General Microbiol..

[27] Lucius S, Makowka A, Michl K, Gutekunst K, Hagemann M (2021). The Entner-Doudoroff pathway contributes to glycogen breakdown during high to low CO2 shifts in the cyanobacterium Synechocystis sp. PCC 6803. Front. Plant Sci..

[28] Selim KA, Lapina T, Forchhammer K, Ermilova E (2020). Interaction of N-acetyl-l-glutamate kinase with the PII signal transducer in the non-photosynthetic alga Polytomella parva: Co-evolution towards a hetero-oligomeric enzyme. FEBS J..

[29] Lapina T, Selim KA, Forchhammer K, Ermilova E (2018). The PII signaling protein from red algae represents an evolutionary link between cyanobacterial and chloroplastida PII proteins. Sci. Rep..

[30] Selim KA, Haffner M, Watzer B, Forchhammer K (2019). Tuning the in vitro sensing and signaling properties of cyanobacterial PII protein by mutation of key residues. Sci. Rep..

[31] Fellers RT (2015). ProSight lite: graphical software to analyze top-down mass spectrometry data. Proteomics.

[32] Benjamini Y, Krieger AM, Yekutieli D (2006). Adaptive linear step-up procedures that control the false discovery rate. Biometrika.

