# Supplementary material for: The redox-sensitive R-loop of the carbon control protein SbtB contributes to the regulation of the cyanobacterial CCM
Source: Sci Rep. 2024 Apr 3;14:7885. doi: 10.1038/s41598-024-58354-7 (PMC10991534; doi:10.1038/s41598-024-58354-7)
Supplement: Supplementary file 1 — Supplementary Information 1. [file 41598_2024_58354_MOESM1_ESM.pdf]

Supplementary material for the manuscript:

The redox-sensitive R-loop of the carbon control protein SbtB contributes to the regulation of the cyanobacterial CCM

Oliver Mantovani[1], Michael Haffner[2], Peter Walke[1], Abdalla A. Elshereef[2,3], Berenike Wagner[2], Daniel Petras[2], Karl Forchhammer[2], Khaled A. Selim[2,4,5*], Martin Hagemann[1,6*]

1 – Institute of Biosciences, Department of Plant Physiology, University of Rostock, Rostock, Germany

2 – Interfaculty Institute of Microbiology and Infection Medicine Tübingen, University of Tübingen, Tübingen, Germany

3 - Chemistry of Natural and Microbial Products Department, Pharmaceutical and Drug Industries Research Institute, National Research Centre, Egypt

4 – Department of Protein Evolution, Max Planck Institute for Biology, Tübingen, Germany

5 - Institute of Biology, Microbiology/Molecular Physiology of Prokaryotes, University of Freiburg, Freiburg, Germany

6 – Interdisciplinary Faculty, Department Life, Light and Matter, University of Rostock, Rostock, Germany

***Corresponding authors:** Khaled A. Selim, Microbiology/ Molecular Physiology of Prokaryotes, Institute of Biology, University of Freiburg, Schänzlestraße 1, 79104 Freiburg, Germany, Email: khaled.selim@biologie.uni-freiburg.de; Martin Hagemann, Institute of Biosciences, Department of Plant Physiology, University of Rostock, A.-Einstein-Str. 3, Rostock D-18059, Germany; Tel: +49(0)3814986110; Fax: +49(0)3814986112; Email: martin.hagemann@uni-rostock.de

Table S1: Primer list

Primers/ amplification	Sequence (5'→3')	Note/ Refs.
**Mutant generation**		
Insertion of AfeI restriction site at the end of *sbtB* coding sequence	**PR1_SbtB_fw:**<br>GGATACACGGTAATGAATACC	[13]
	**PR2_SbtB_AfeI-STOP_rv:**<br>CCTCAGGTCATTAAGCGCTGAAAGTATGCCCATAAAGTACTTC	[13]
Mutants verification	**2059:**<br>GTTATTTTGTCGTCAAAC	[13]
	**2092:**<br>GGATCCCCAATAGTTTATGGTC	[13]
	**#PR6_SbtB_pUC19_seq_rv:**<br>CTGCCCTGCTGCGTAACATCGTTGC	This study
**Recombinant proteins**		
C-terminal StrepII-tagged *Sc*SbtB (*slr1513*); (pASK-IBA3_*Sc*SbtB-strep plasmid)	1256_Fw:<br>GTGAAATGAATAGTTCGACAAAAATCTAGATAACGAGGGCAAAAAATG<br>GCTAAACCAGCGAACAAGCTCG	[11]
	1257_Rv:<br>AAGCTTATTATTTTTCGAACTGCGGGTGGCTCCAAGCGCTACAGCCCT<br>CAGGGCCACAGAAAG	[11]
C-terminal StrepII-tagged *Sc*SbtB-Δ104 (pASK-IBA3_*Sc*SbtB-Δ104)	1256_Fw:<br>GTGAAATGAATAGTTCGACAAAAATCTAGATAACGAGGGCAAAAAATGGCTAAACCAGCGAACAAGCTCG	[13]
	1663_Rv_SbtB delta 104:<br>CAAGCTTATTATTTTTCGAACTGCGGGTGGCTCCAAGCGCTGAAAGTATGCCCATAAAGTACTTCTGC	[13]
C-terminal StrepII-tagged *Sc*SbtB-Δ109 (pASK-IBA3_*Sc*SbtB-Δ109)	1256: Slr1513_Fw:<br>GTGAAATGAATAGTTCGACAAAAATCTAGATAACGAGGGCAAAAAATGGCTAAACCAGCGAACAAGCTCG	This study
	1661: Rv_SbtB W/o C 110:<br>AAGCTTATTATTTTTCGAACTGCGGGTGGCTCCAAGCGCTGCCCTCAGGGCCACAGAAAG	This study
C-terminal StrepII-tagged *Sc*SbtB-C105S+C110S (pASK-IBA3_*Sc*SbtB-C105S+C110S)	1256_Fw:<br>GTGAAATGAATAGTTCGACAAAAATCTAGATAACGAGGGCAAAAAATG<br>GCTAAACCAGCGAACAAGCTCG	[13]
	1761_Rv_SbtB-C105+110S<br>CAAGCTTATTATTTTTCGAACTGCGGGTGGCTCCAAGCGCTGCTGCCCTCAGGGCCGCTGAAAGTATGCCCATAAAGTACTTCTGC	[13]
C-terminal StrepII-tagged *Sc*SbtB-C105A+C110A (pASK-IBA3_*Sc*SbtB-C105A+C110A)	1256_Fw:<br>GTGAAATGAATAGTTCGACAAAAATCTAGATAACGAGGGCAAAAAATG<br>GCTAAACCAGCGAACAAGCTCG	[13]
	1759_Rv_SbtB-C105+110A<br>CAAGCTTATTATTTTTCGAACTGCGGGTGGCTCCAAGCGCTTGCGCCCTCAGGGCCTGCGAAAGTATGCCCATAAAGTACTTCTGC	[13]
N-terminal StrepII-tagged *Sc*SbtB-WT (pASK-IBA5_*Sc*SbtB-WT)	1530: FW:_SbtB-Native- N ST:<br>GAGCCACCCGCAGTTCGAAAAAGGCGCCGACGACGACGACAAGATGGCTAAACCAGCGAACAAGCTC	This study
	1531: Rv_SbtB-Native_N ST:<br>CGACCTCGAGGGATCCCCGGGTACCGAGCTCGAATTCGGGACCTTAACAGCCCTCAGGGCCACAG	This study
C-terminal StrepII-tagged *Sc*SbtB-C105S (pASK-IBA3_*Sc*SbtB-C105S)	1256: Slr1513_Fw:<br>GTGAAATGAATAGTTCGACAAAAATCTAGATAACGAGGGCAAAAAATGGCTAAACCAGCGAACAAGCTCG	This study
	1760: RV_SbtB C-105-S:<br>CAAGCTTATTATTTTTCGAACTGCGGGTGGCTCCAAGCGCTACAGCCCTCAGGGCCGCTGAAAGTATGCCCATAAAGTACTTCTGC	This study
C-terminal StrepII-tagged *Sc*SbtB-C110S (pASK-IBA3_*Sc*SbtB-C110S)	1256: Slr1513_Fw:<br>GTGAAATGAATAGTTCGACAAAAATCTAGATAACGAGGGCAAAAAATGGCTAAACCAGCGAACAAGCTCG	This study
	1662: Rv_SbtB-C110S:<br>AAGCTTATTATTTTTCGAACTGCGGGTGGCTCCAAGCGCTGCTGCCCTCAGGGCCACAGAAAG	This study

N-terminal His_6-tagged TrxA (*slr0623*); (pET15b_TrxA-His_6 plasmid)	pET15b_slr0623_fw: CAGCAGCGGCCTGGTGCCGCGCGGCAGCCATATGCTCGAGATGAGTGCTACCCCTCAAGTTTC	[13]
	pET15b_slr0623_rev: CCCTCAAGACCCGTTTAGAGGCCCCAAGGGGTTATGCTAGTTATTGCTCAGCGGTGGCAGCAGCCAAC	[13]

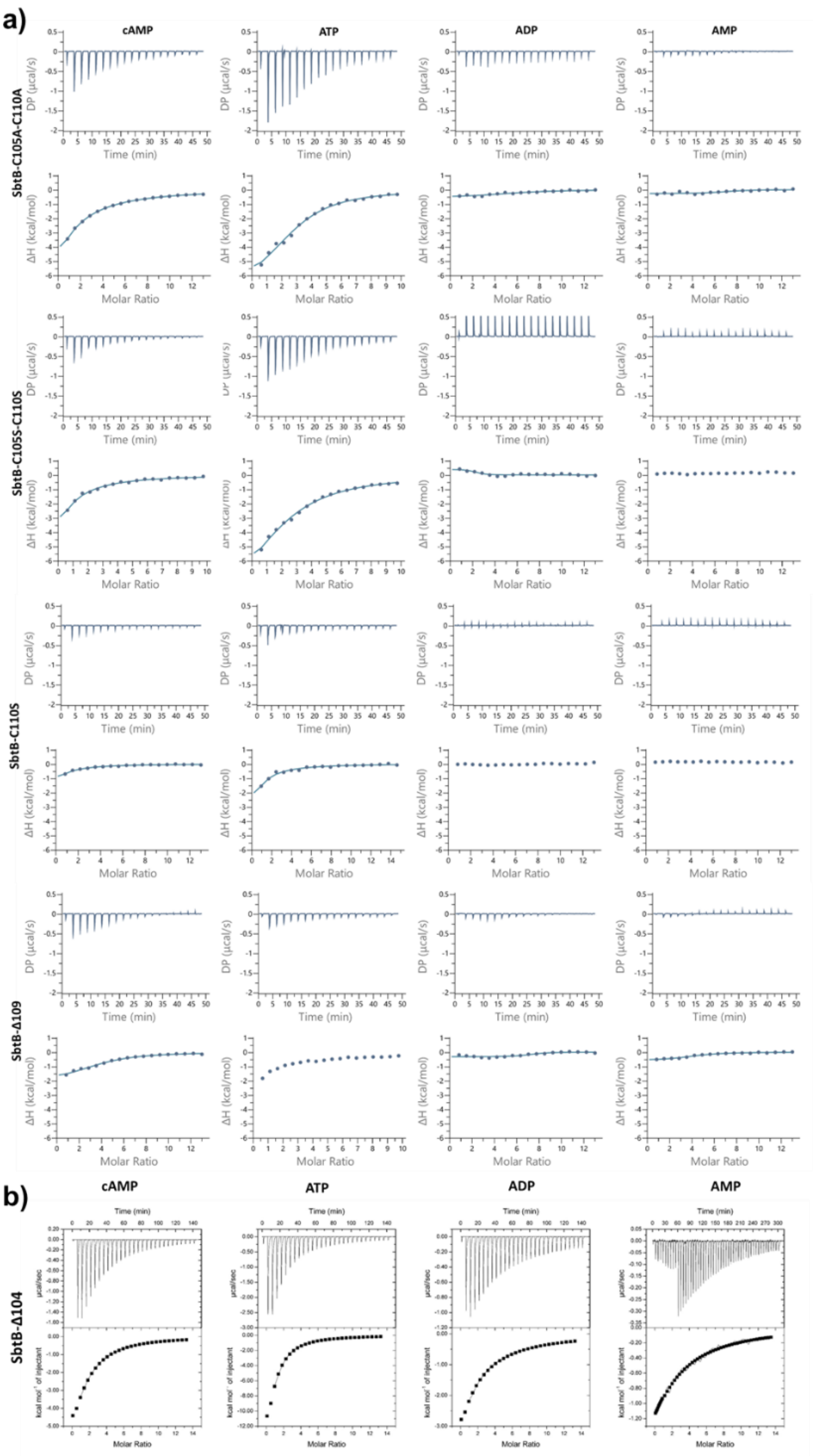

Figure S1: Isothermal titration calorimetry (ITC) analysis of ligand binding properties of the SbtB R-loop variants. a) Upper panels show the raw ITC data in the form of the heat produced during the titration of wild-type and site-specific variant SbtB (trimeric concentration), respectively, with different effector molecules; lower panels show the binding isotherms and the best-fit curves according to the one binding site models for trimeric SbtB. b) ITC analysis of ligand binding properties of the SbtB R-loop variant (Δ104).

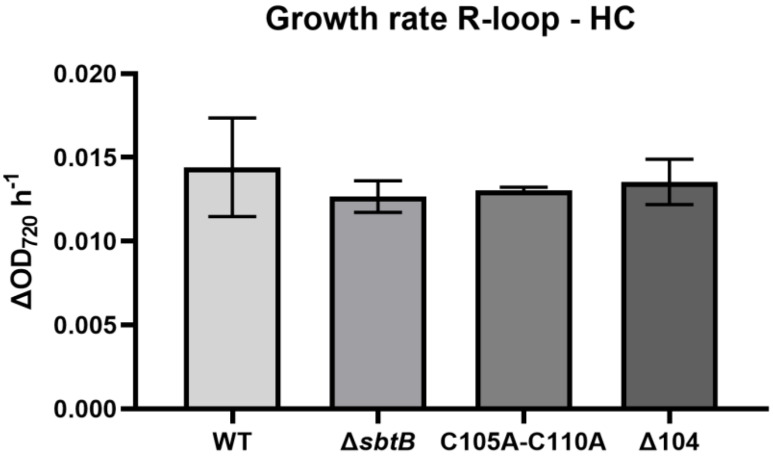

***Figure S2: Growth rate analysis of* Synechocystis *R-loop mutants under HC conditions*.** Growth of strains wild type (WT), Δ*sbtB*, C105A-C110A, and Δ104 was analyzed at high CO_2 (5%, HC) and continuous light in the Multicultivator MD1000. The growth rate (ΔOD_{720}/h) was determined during the exponential phase.

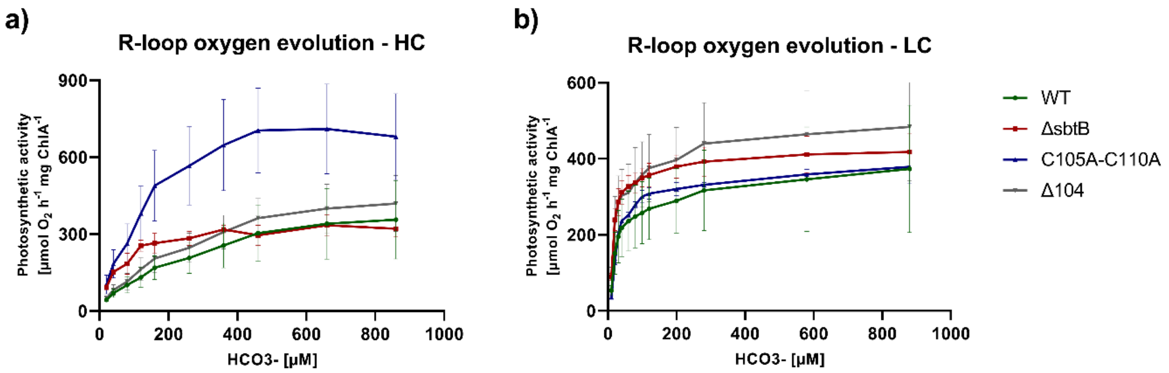

Figure S3: Bicarbonate-dependent oxygen evolution analysis of **Synechocystis *R-loop mutants*.** Bicarbonate-dependent oxygen production via photosynthesis was measured for strains wild type (WT), Δ*sbtB*, C105A-C110A, and Δ104 that were acclimated to either high CO_2 (5%, HC) (**a**) or ambient CO_2 (0.04%, LC) (**b**) conditions.

Sample A): SbtB C-terminal tagged (oxidized)
Experimental Monoisotopic Mass: 13063.55

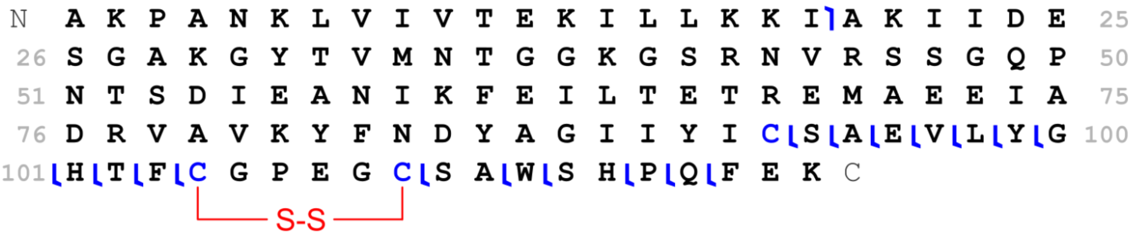

Sample B.1): SbtB C-terminal tagged (DTT reduced)
Experimental Monoisotopic Mass: 13063.57

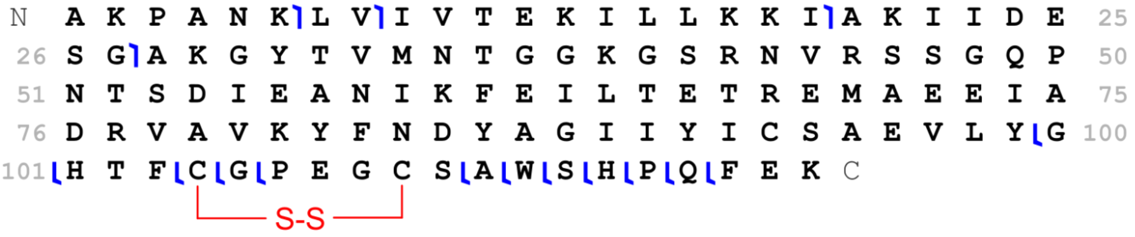

Sample B.2): SbtB C-terminal tagged (DTT reduced)
Experimental Monoisotopic Mass: 13065.56

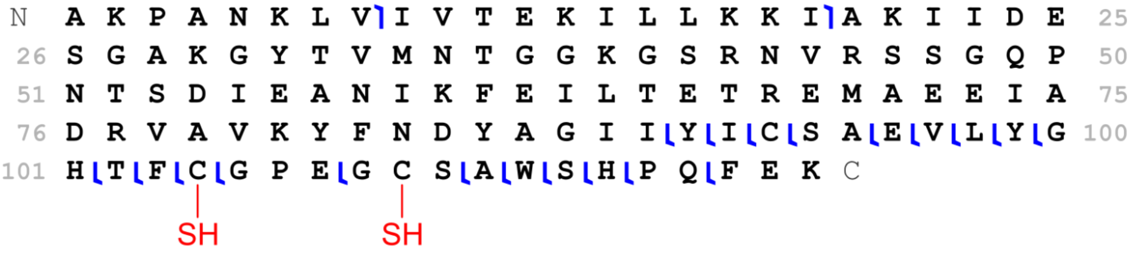

Sample C): SbtB C-terminal tagged (DTT-TrxA-reduced)
Experimental Monoisotopic Mass: 13065.59

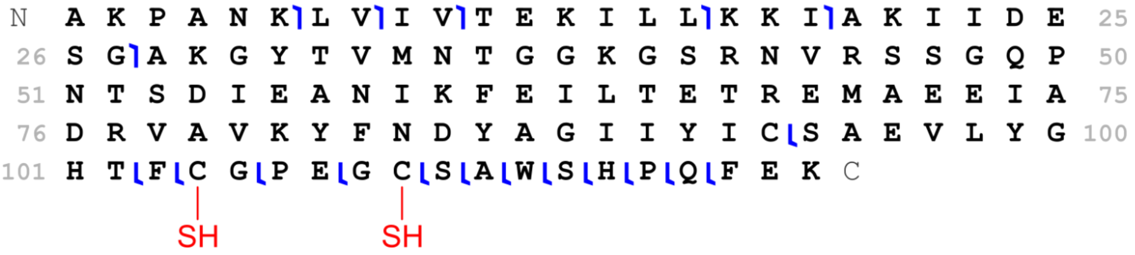

Figure S4: Peptide sequence of oxidized and reduced SbtB. Peptide fragment maps were generated with the MS/MS data shown in Fig. 5. **A-C** indicate the presence of a disulfide bond in (**Sample A**; black peaks in Figure 5A) and the absence of such bond in (**Sample C**; red peak in Figure 5 C). **Sample B** contained SbtB in both oxidized and reduced states.

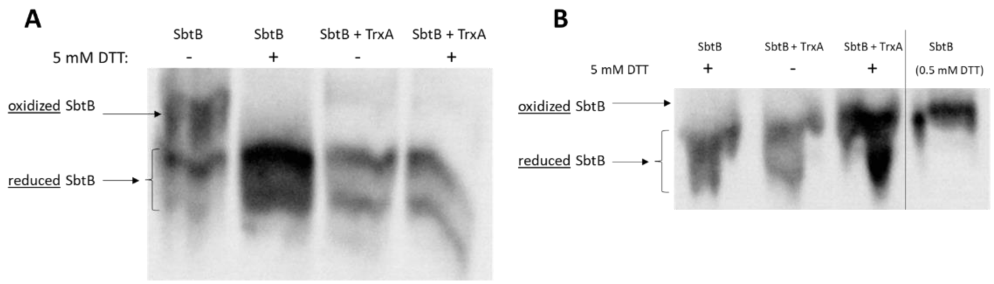

Figure S5: Effect of TrxA and/or DTT additions on reduction of SbtB, resolved on Urea-PAGE and detected using SbtB antibody. Urea-PAGE gel showing the reduction of SbtB by 5 mM DTT or equimolar TrxA concentrations (**A**), compared to 0.5 mM DTT (**B**). The full-size Western-blots are shown in Suppl. Fig. S8.

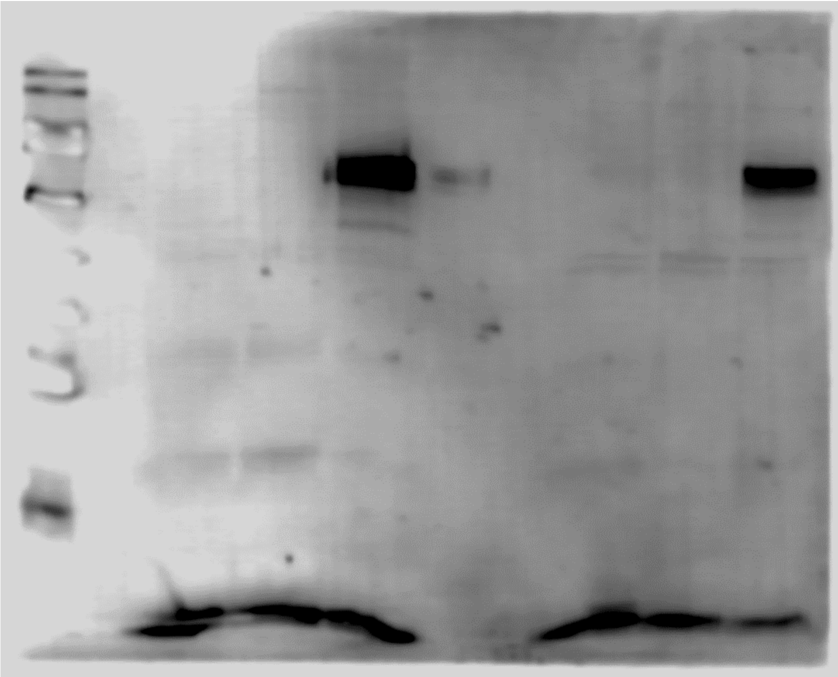

***Figure S6: Full-size Western blot of figure 6a*.** Please note that the high molecular mass signals most probably represent stable TrxA/SbtB complexes.

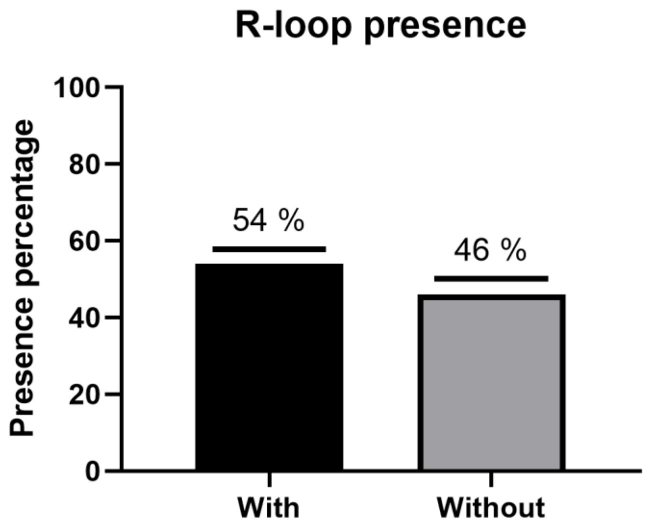

***Figure S7: Presence of the R-loop with the highly conserved cysteine residues among cyanobacterial SbtB homologs*.** R-loop presence percentage was calculated from a multiple sequence alignment of 460 SbtB sequences (sequences obtained from the NCBI data base in June 2023, alignment done with ClustalW).

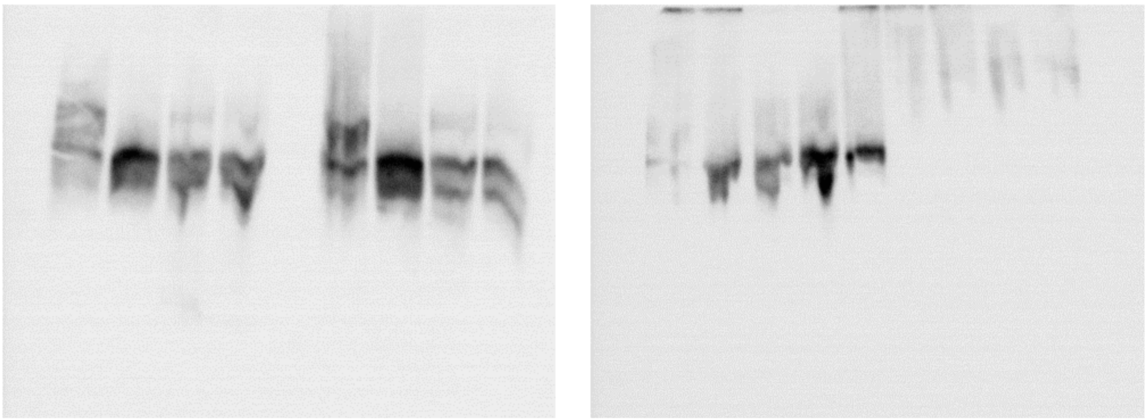

***Figure S8: Full-size Western blots of Suppl. figure S5*.**
