# Supplementary figures and images for: The redox-sensitive R-loop of the carbon control protein SbtB contributes to the regulation of the cyanobacterial CCM
Source: Sci Rep. 2024 Apr 3;14:7885. doi: 10.1038/s41598-024-58354-7 (PMC10991534; doi:10.1038/s41598-024-58354-7)

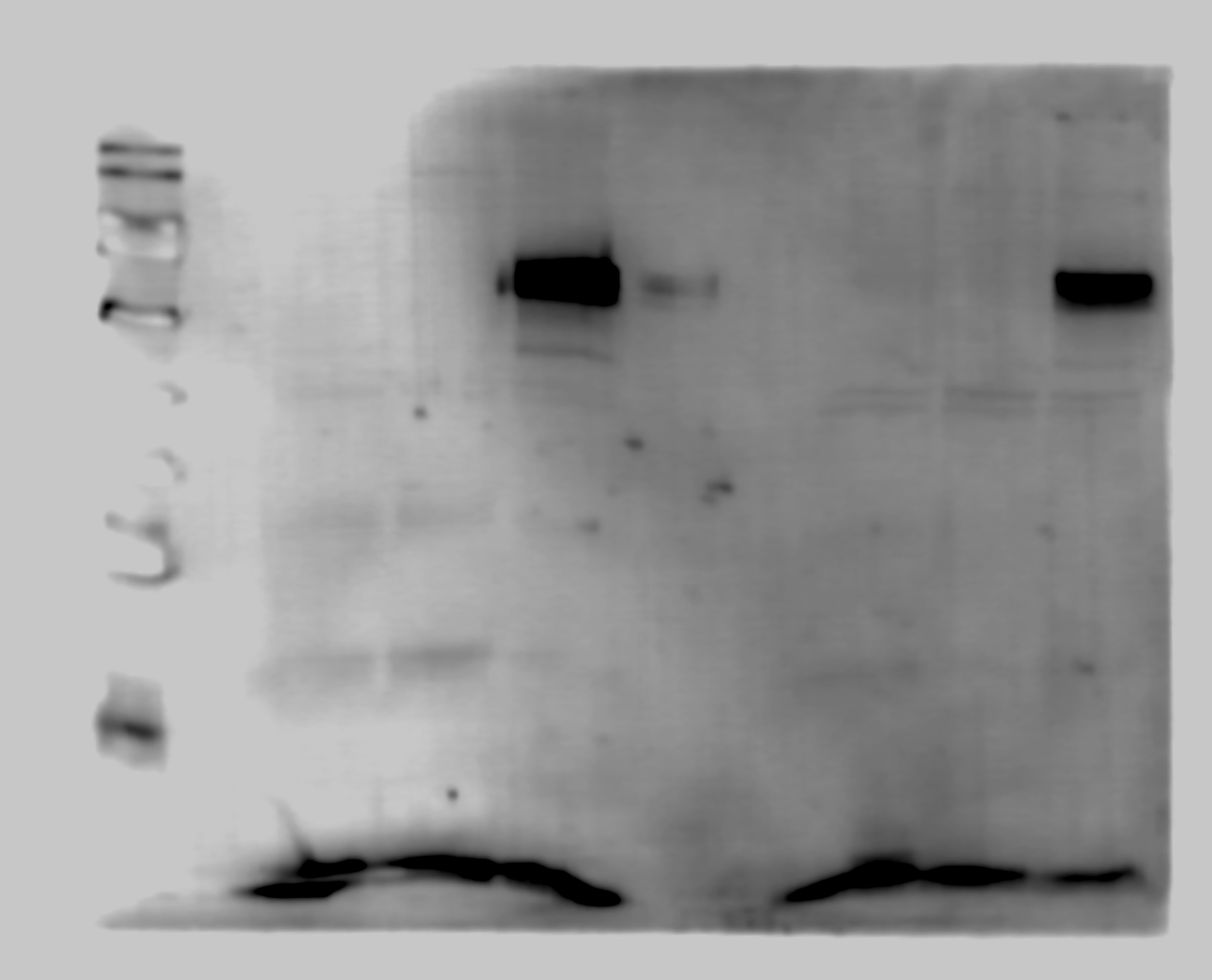

Supplement: Supplementary file 4 — Supplementary Information 4. [file 41598_2024_58354_MOESM4_ESM.tif]
